# Understanding the Intention-to-treat Principle in Randomized Controlled Trials

**DOI:** 10.5811/westjem.2017.8.35985

**Published:** 2017-09-18

**Authors:** C. Eric McCoy

**Affiliations:** University of California, Irvine, School of Medicine and Medical Center, Department of Emergency Medicine, Orange, California

## Abstract

Clinicians, institutions, and policy makers use results from randomized controlled trials to make decisions regarding therapeutic interventions for their patients and populations. Knowing the effect the intervention has on patients in clinical trials is critical for making both individual patient as well as population-based decisions. However, patients in clinical trials do not always adhere to the protocol. Excluding patients from the analysis who violated the research protocol (did not get their intended treatment) can have significant implications that impact the results and analysis of a study.

Intention-to-treat analysis is a method for analyzing results in a prospective randomized study where all participants who are randomized are included in the statistical analysis and analyzed according to the group they were originally assigned, regardless of what treatment (if any) they received. This method allows the investigator (or consumer of the medical literature) to draw accurate (unbiased) conclusions regarding the effectiveness of an intervention. This method preserves the benefits of randomization, which cannot be assumed when using other methods of analysis.

The risk of bias is increased whenever treatment groups are not analyzed according to the group to which they were originally assigned. If an intervention is truly effective (truth), an intention-to-treat analysis will provide an unbiased estimate of the efficacy of the intervention at the level of adherence in the study. This article will review the “intention-to-treat” principle and its converse, “per-protocol” analysis, and illustrate how using the wrong method of analysis can lead to a significantly biased assessment of the effectiveness of an intervention.

The most effective way to establish a causal relationship between an intervention and outcome is through a randomized controlled trial (RCT) study design.^1^–^3^ Randomization affords an unbiased comparison between groups as it controls for both known and unknown confounding variables. If done correctly, randomization yields groups that are balanced with regard to prognostic variables (variables that have an impact or an influence on developing the outcome under study). If two (or more) groups are prognostically balanced, with the exception of the intervention, and an investigator observes a difference in outcomes, a sound argument can be made attributing the difference in result to the intervention under study.

Although recognized as the “gold standard” study design for establishing a causal relationship between intervention and outcome, the process of randomization alone does not wholly guard against bias. Incorrect analysis of the data can introduce bias even in the setting of the correct implementation of a valid random allocation sequence. It is therefore important to preserve the integrity of randomization during the implementation of the study and in analysis. One such way investigators and consumers of the medical literature may arrive at an incorrect and biased assessment of results is by failing to evaluate patients according to the group to which they were originally assigned.

Anything that disrupts the prognostic balance afforded by randomization introduces bias into the study and analysis. Therefore, the goal of the investigator is to preserve this prognostic balance throughout the entire study, including the analysis phase after all data and outcomes have been recorded. The concept of analyzing patients according to which group they were originally assigned is called intention-to-treat analysis (or the intention-to-treat principle).^4^,^5^ In this article, the author presents and reviews a hypothetical example to illustrate how failure to apply this concept when interpreting results from a randomized trial can lead to misleading conclusions.

## Example illustrating importance of intention-to-treat principle in an RCT

Imagine an investigator wants to evaluate whether adding a surgery to conventional medical therapy (medical management + surgery = intervention) is effective for preventing death (outcome) in patients with cardiovascular disease (Figure). Two hundred patients are enrolled in an RCT, 100 of whom are allocated to each arm. In this example, group A receives the intervention (medical management + surgery) and group B serves as the active control (medical management only). All outcomes are evaluated after 12 months.

In this example, there is a six-week waiting period between randomization and surgery. In group A, 30 total patients have died (the primary outcome of the trial) at the 12-month follow-up. Of these 30 patients, 15 died within three weeks after enrollment and the remaining 15 died between six weeks and 12 months. The patients in group B have a similar outcome: 30 total patients have died at the 12-month follow-up. Of these 30, 15 died within three weeks after enrollment and the remaining 15 died between six weeks and 12 months (Figure).

Let’s also assume that the surgical intervention has no effectiveness, no impact on the primary outcome (death), and we will call this “truth.” Investigators conduct randomized trials to discover the “truth” as to whether or not an intervention is effective. Our unbiased assessment of the study results (our search for truth) will depend on how we analyze the data. If analyzed correctly, we should come to the conclusion that the surgical intervention is ineffective, and if analyzed incorrectly, we will arrive at a spurious, biased conclusion that the surgery is effective.

## PER-PROTOCOL ANALYSIS

We will begin our analysis according to who actually received the intervention assigned by the protocol. This method of analyzing the data is called per-protocol analysis, also referred to as efficacy, explanatory analysis, or analysis by treatment administered.^4^ For the intervention group (A), 85 patients actually received the intervention, as 15 patients died before they had the opportunity to undergo surgery. The risk of death according to this method of analysis is 0.18 or 18% (15/85). For the control group (B), the risk of death is 0.3 or 30% (30/100) (Figure).

The risk of death in the intervention group (A) compared to the risk of death in the control group (B) is called the relative risk (RR). This is calculated by taking the ratio of the two risks, in this case 0.18/0.3. Doing the math yields a relative risk of 0.59 or 59%. The relative risk reduction of death can be calculated by subtracting the relative risk from 1 (when RR is expressed as a proportion). In this example, that would yield 0.41, or 41% (1 – 0.59).

So analyzing the data according to a per-protocol analysis would lead an investigator (or consumer of the medical literature) to spuriously conclude that the intervention (medical management + surgery) reduces the risk of death by 41% when compared to conventional therapy (medical management) alone. However, as discussed before, we know that surgery in this example has absolutely no effect on the outcome (truth). This method of analysis would result in a gross misinterpretation and inaccurate (biased) assessment of the effectiveness of the intervention.

Even more alarming would be the application of this inaccurate interpretation to clinical practice, where patients would be subject to an intervention with no benefit but with associated risks. A distinct but related type of analysis where patients are analyzed according to the treatment they actually received (regardless of their originally assigned group) also introduces bias into the analysis of a randomized study by disrupting the prognostic balance created by randomization. This method of evaluating patients according to which treatment they actually received is called as-treated analysis.^3^ In this method, if a patient in the control group received surgery (regardless of the reason), they would be analyzed in the intervention group, and vice versa. Both per-protocol and as-treated analyses increase the risk of bias when evaluating the results of a RCT. Fortunately, for investigators and consumers of the medical literature, there is a method to analyze data from a randomized trial that will not lead to this type of spurious conclusion. This method is called intention-to-treat analysis.

## INTENTION-TO-TREAT ANALYSIS

Intention-to-treat analysis analyzes the patients according to the groups to which they were originally assigned. A process that has once been described as “once randomized, always analyzed” reminds us to always analyze patients according to their original group assignment. This method of analysis preserves the prognostic balance afforded by randomization. In this example, the risk of death for the intervention group (A) is 0.3 or 30% (30/100). Using this method of analysis, the 15 patients who died (the primary outcome of the study) before they were to get the intervention are included in the calculation. For the control group (B), the risk of death is 0.3 or 30% (30/100).

The relative risk for death in patients receiving the intervention compared to the control group is 1 (0.3/0.3). And the relative risk reduction is 0 (1–1). So analyzing the data according to the intention-to-treat principle correctly concludes that the surgical intervention does not work. Some would argue, “Is it fair to include the 15 patients who died before receiving the intervention (medical management + surgery)?” Yes. Removing patients from either arm of the study disturbs the prognostic balance afforded by randomization. Although with few exceptions, excluding patients from a randomized trial will increase the risk of bias in a study.^6^,^7^ Theoretically, the only way patients can be lost from a study and not increase the risk of bias is if the patients who are lost are prognostically identical to the patients who remain.

However, research has shown that patients who do not adhere to the treatment assigned differ in ways more than just their adherence. Empirical evidence suggest that participants who adhere tend to do better than those who do not adhere, regardless of assignment to active treatment or placebo and even after adjustment for all known prognostic factors.^4^,^8^,^9^ In a prospective placebo controlled trial evaluating the effectiveness of a lipid-lowering agent to reduce mortality in men suffering a myocardial infarction, the investigators observed a significant increase in mortality in nonadherent patients when compared to adherent patients, regardless of whether they received the intervention drug or placebo.^8^ A meta-analysis evaluating the relationship between adherence to drug therapy and mortality concluded that adherence to drug therapy is associated with positive health outcomes.^9^ The authors also report that the observed association between good adherence to placebo and decreased mortality supports the existence of the “healthy adherer” effect, whereby adherence to drug therapy may be a surrogate marker for overall health behavior.^9^ The intention-to-treat analysis preserves the prognostic balance afforded by randomization, thereby minimizing any risk of bias that may be introduced by comparing groups that differ in prognostic variables.

Applying the intention-to-treat principles yields an unbiased estimate of the efficacy of the intervention on the primary study outcome at the level of adherence observed in the trial. So in the instance when the treatment under study is effective, but there is substantial nonadherence, the intention-to-treat analysis will underestimate the magnitude of the treatment effect that will occur in adherent patients. Although an underestimate of an effective therapy, it will be unbiased. This method of analysis results in a more accurate, unbiased estimate than that yielded from a per-protocol or as-treated type of analysis.

## Figures and Tables

**Figure:**
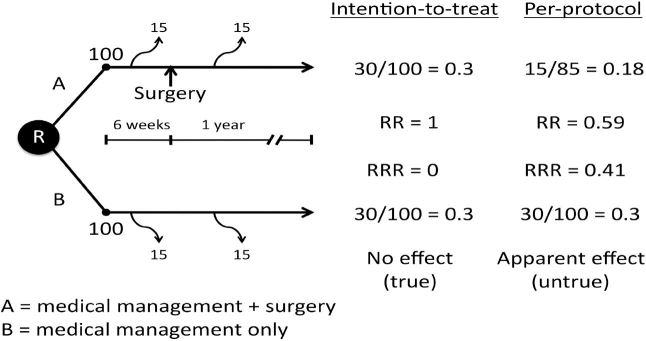
Hypothetical prospective randomized controlled trial evaluating effectiveness of intervention (A = medical management + surgery) vs. control (B = medical management only) in patients with cardiovascular disease. *R*, randomization; *RR*, relative risk; *RRR*, relative risk reduction.
